# Supplementary material for: Are carbon emissions trading and green financial instruments synergistic? -Comprehensive quantitative research based on content analysis
Source: PLoS One. 2024 Mar 7;19(3):e0298601. doi: 10.1371/journal.pone.0298601 (PMC10919601; doi:10.1371/journal.pone.0298601)
Supplement: S1 Data — (ZIP) [file pone.0298601.s001.zip › Data/地级市Z_F工作报告.zip!/╝¬┴╓/┴╔╘┤╩╨/└┤╫╘╣1⁄2╓┌║┼í╛┬φ┐╦╩2╛▌═°í┐_2013.docx]

各位代表：

　　现在，我代表市政府，向大会作政府工作报告，请予审议，并请市政协各位委员提出意见。

　　一、过去五年工作回顾

　　过去的五年，是我市发展史上极不平凡的五年。在省委、省政府和市委的正确领导下，市政府深入贯彻落实科学发展观，突出发展第一要务、民生第一目标、稳定第一责任，凝心聚力，开拓进取，全面实现了十一五规划目标和十二五良好开局，圆满完成了市六届人大一次会议确定的各项任务，开创了辽源转型发展新局面。

　　综合实力大幅提升。预计2012年，地区生产总值突破600亿元，全口径财政收入达到40亿元，分别是2007年的2.8倍和2.9倍。全社会固定资产投资完成520亿元，社会消费品零售总额达到150亿元，外贸进出口总额实现1.3亿美元，分别是2007年的4.7倍、2.5倍和3.1倍。工业经济快速增长，规模以上工业总产值超过1000亿元，是2007年的4.4倍。农业综合生产能力不断提高，粮食总产量达到43亿斤。新兴服务业态不断涌现，金融、信息、商贸物流、文化旅游等产业快速发展。民营经济日趋活跃，主要指标增幅连续三年位居全省前列。我市城市综合增长竞争力连续三年位列全国294个地级市前五位。

　　经济转型成效显著。累计实施投资3000万元以上项目968个，其中亿元以上项目467个。新材料、新能源、医药健康、装备制造、冶金建材、轻工纺织等接续替代产业迅速形成规模，占工业比重达到78.6%。高精铝加工、纺织袜业技术装备达到国际先进水平。辽源经济开发区启动二次创业，产业层次不断提高，经济增长极作用日益凸显，已经跻身全省省级开发区前十位。各县（区）工业集中区实现升级晋位，承载项目能力明显增强。科技创新步伐加快，高科技产业增加值占比达到25%，新产品产值率达到21.5%。节能减排力度加大，万元GDP能耗累计下降23.6%，COD、二氧化硫分别减排0.6万吨和3.4万吨。我市转型工作顺利通过国家中期评估，并被誉为辽源样本。

　　城乡面貌焕然一新。确立了山水林城城市转型理念，形成了具有辽源特色的城乡建设规划体系。启动了南部新城、寿山新区、辽白经济一体化等重大工程，两县城区和重点镇建设加快推进。新农村建设成效明显。全市城镇化率达到58.4%。辽伊、营东梅、吉草高速和辽西铁路建成通车，域内高等级公路里程达到329公里，农村公路实现村村通。完成杨木水库等25座中小型水库除险加固和东辽河城区段综合治理等一批重点水利工程，城市防洪、供水能力明显提高。大唐热电二期工程并网投产。全省首个智能电网加快建设。天然气管网覆盖主城区。新建、改造城区主干路37条、巷路149条、桥梁12座，城市路网体系日趋完善。建成仙城大街景观带、向阳山森林公园、辽河广场等一批公共设施。五城联创取得积极成果，省级园林城一年完成创建，省级卫生城两年通过验收，省级生态城、国家级环保模范城和国家级文明城创建工作扎实推进。

　　人民生活明显改善。连续五年实施惠民实事行动计划，新增财力70%以上用于民生投入。集中连片改造棚户区34片，建设保障房9.8万套，6.7万户居民喜迁新居。全面完成采煤沉陷区综合治理和农村泥草房改造任务。实施既有建筑节能改造336万平方米，改造老旧小区115个。解决16万农村人口饮水安全问题。开发公益性岗位7355个,零就业家庭动态为零，城镇登记失业率控制在3.9%以内。深入推进全民创业，东北袜业园被命名为全国创业孵化示范基地，我市被国务院评为全国创业先进城市。城镇居民人均可支配收入和农民人均纯收入达到21570元和9153元，年均递增15%和21%。城乡低保标准、养老和失业保险待遇逐年递增，率先在全省实现新农保、城居保地区全覆盖。城市公共交通加快发展，群众出行条件明显改善。殡仪服务中心收归公营，减免低保困难家庭六项殡葬费用，居民殡葬支出大幅降低。全民健身中心等一批文体设施交付使用。各类教育协调发展。医疗卫生服务体系基本形成。出生人口素质明显提高。

　　改革开放不断深化。政府机构、街道社区、文化体制、国有粮食购销企业和集体林权制度改革基本完成，事业单位分类改革、行政审批权相对集中改革、医药卫生体制改革、统计体制改革扎实推进，投融资体制改革、城镇化配套改革和民营企业股份制改造不断加快。招商引资领域拓展到欧洲、北美、东南亚、长三角、珠三角等40多个国家和地区，累计签约项目400余个，引进域外资金1180亿元，实际利用外资7.8亿美元。香港中聚、大连美罗、宁波均胜、长春欧亚等知名企业落户辽源。铝型材、鞋袜省级出口基地加快建设，全市有23户企业、53种产品打入国际市场。规范政府融资平台，搭建行业融资平台，成功举办三届PE走进辽源活动，累计融资126亿元，发行企业债20亿元。辽源郊区联社改制为农商行。麦达斯控股、利源铝业成功上市。得亨公司完成资产重组，实现一业变两业。

　　和谐局面持续巩固。建立人民调解、行政调解、司法调解三位一体大调解工作体系，创新交通事故处理、医疗纠纷调解和街道社区一体化服务模式，实现了街道社区化、社区网格化、网格精细化管理。扎实开展积案化解年和局长大接访活动，社会矛盾调解成功率、信访积案化解率分别达到98%和94%。扎实推进平安辽源建设，构筑公共安全防控体系，刑事案件、治安案件连续三年下降，群众安全感满意度处于全省前列，连续两年荣获全国见义勇为城市奖。始终保持安全生产高压态势，各类事故逐年下降，连续三年未发生较大以上事故，煤矿安全生产超过1200天。连续28年无重大森林火灾。民族团结、宗教和谐局面进一步巩固。国防后备力量建设质量不断提升，军地联合应对重大突发事件机制基本建立。食品药品安全监管、突发事件应急处置、城市管理综合行政执法工作体系初步形成。

　　民主法制和政府自身建设全面加强。自觉接受人大法律监督、工作监督和政协民主监督，充分听取社情民意，代表建议、政协提案办理效率不断提高。坚持科学民主决策，健全完善决策咨询、公众参与、责任追究和社会风险评估制度。全面开展行政审批大提速、遏制行政乱处罚工作，实行企业宁静日制度，大力推进行政服务便利化，全省软环境评价我市升至第二位。严格落实领导干部一岗五责，建立重点工作绩效考评和重大项目推进机制。加强政府廉政建设，突出重点领域和源头控制，充分发挥监察、审计等部门职能作用，确保行政权力阳光运行。

　　外事侨务、人防、气象、地震、档案、方志及妇女儿童、老龄、残疾人等各项事业取得新进步。

　　各位代表，即将过去的2012年，面对复杂严峻的国内外经济形势，市政府认真贯彻市第六次党代会作出的战略部署，紧紧围绕加快发展、全面转型，突出招商引资和项目建设总抓手，上争下促，攻坚克难，经济社会发展保持良好势头。一是抓经济平稳增长。强化工业对经济的支撑作用，积极配置生产要素，帮助停产减产企业恢复正常生产，支持重点骨干企业做大做强。产值超亿元企业达到200户，产值超10亿元企业达到13户。调整优化投资结构，加快培育现代服务业，促进建筑、房地产业健康发展，积极培育新的经济增长点。受宏观经济下行影响，虽然地区生产总值、全口径财政收入等指标增幅低于年初预期，但高于全省平均水平，全社会固定资产投资、社会消费品零售总额、外贸进出口总额、民营经济主营业务收入等指标增幅居全省前列。二是抓重大项目支撑。组织实施投资3000万元以上重点项目335个，其中10亿元以上项目32个。鑫达钢铁二期、利源铝业三期、东北袜业二期、方大锻造、均胜汽车零部件等工业项目快速推进；辽河北岸城市综合体、凯旋王国创意园、茵特拉根欢乐广场等现代服务业项目进展顺利；东辽河流域综合治理、国家矿山湿地公园等生态环保项目启动建设；富奥泵业异地建设、鸿图纸业锂电池隔膜纸等130个重点项目竣工或投产。辽源经济开发区发展空间不断拓展，东丰经济开发区升级为省级开发区，东辽工业集中区承载功能日趋增强，龙山、西安工业集中区建设取得突破。三是抓招商引资攻坚。签约重点项目146个，其中10亿元以上项目26个，引进域外资金420亿元，实际利用外资2亿美元，分别比上年增长1.5倍和1.3倍。深圳中意、深圳海王、IBM、AGT等知名企业相继落户辽源，与东南亚、长三角、珠三角等重点区域政府机构、行业协会和大型企业联系日趋紧密。全市招商引资氛围日益浓厚，项目层次、招商实效显著提升。四是抓城市建设提速。南部新城四路四桥竣工通车，四馆、工商学校主体封闭。辽白经济一体化加快推进，辽源与白泉实现公交、电讯同城化。辽西高速开工建设。市区九路一桥工程完工，仙城客运站、公交客运枢纽中心和兴国电动汽车充电站启动运营。城区植树20万株，新增绿地21.8万平方米。实现三山、两街、一河景观亮化。污水处理厂完成升级改造。博纳电影城投入使用。大力推进五城联创，市容环境、交通秩序明显改善，占道经商、违章建筑问题得到一定遏制，城市管理综合行政执法力度不断加大。成功列入全国首批智慧城市试点，13家国内外知名软件企业落户辽源，IBM东北首家卓越云计算中心启动运营。五是抓保障性安居工程。多方筹措资金，实施合力、和谐征收，当年征收房屋1.4万户，开工建设保障房32711套，回迁居民1.5万户，富国、丘下等重点区片棚户区改造顺利推进。完成既有建筑节能改造236万平方米，改造老旧小区74个，托管弃管单体楼125栋，黑楼道问题基本解决。六是抓和谐辽源、活力辽源建设。创新社会管理方式，组建群众工作专门服务机构，集中开展矛盾调处、群众信访、投诉受理、法律援助等工作。强化信访积案化解攻坚，国家和省交办的涉法涉诉案件全部结案。推进特色文化名城建设，公共文体设施不断完善，群众性文化活动蓬勃开展，成功举办首届国际琵琶文化艺术节、国际袜业交易会、东丰农民画艺术节、东辽民俗文化艺术节，荣获全国文化体制改革工作先进地区称号。

　　各位代表，过去的五年，是辽源经济实力大幅跃升、知名度和影响力不断扩大的五年；是城市功能品位提升、城乡面貌变化显著的五年；是民生不断改善、百姓得到更多实惠的五年；是推进全面转型、科学发展迈出坚实步伐的五年。五年来，全市广大干部群众团结拼搏，锐意进取；广大企业家勇担责任，追求作为；社会各界广泛参与，和衷共济；市人大、市政协和各民主党派有效监督，鼎力支持。勤劳智慧的辽源人民用激情和汗水谱写了不畏艰难、追求卓越的辉煌篇章。在此，我代表市政府，向各民主党派、无党派人士、人民团体，向解放军驻辽部队、武警官兵，向各级老领导、老同志，向中省直单位和来辽投资者，向全市各族人民以及所有关心、支持我市经济社会发展的各界人士，表示衷心的感谢并致以崇高的敬意！

　　各位代表，五年的转型成果鼓舞人心，五年的发展经验弥足珍贵。工作中我们深切感到：必须坚持党的领导，把握中心，凝聚人心，始终保持目标清晰和方向正确；必须坚持科学发展、加快发展，理性把握客观经济规律，始终从人民迫切需要和辽源实际出发；必须坚持不断解放思想，以理念更新引领实践创新，始终用发展的办法解决前进中的问题；必须坚持以人为本，突出人民群众的主体地位，始终把保障和改善民生作为一切工作的出发点和落脚点；必须坚持改革开放，强化招商引资和项目建设核心之举，始终为辽源转型发展注入不竭动力；必须坚持效率与公平并重，更加注重维护社会公平正义，始终营造稳定和谐的良好社会环境。

　　肯定成绩的同时，我们更要清醒看到,　辽源加快发展、全面转型的任务仍然艰巨繁重，诸多困难和问题亟待有效破解。主要是：经济总量偏小、产业结构不优、发展基础薄弱仍是基本市情；经济增长质量不高，财政支撑能力不强，投入产出周期过长，资源环境约束加剧；居民收入增长不快，公共服务能力有待提高，一些关系人民群众切身利益的问题解决得还不够好；制约科学发展的体制机制障碍仍然存在，机关行政效能、干部素质能力有待全面提升，软环境建设还需进一步加强，等等。对此，我们必须高度重视，采取有力措施，切实加以解决。

　　二、今后五年目标任务

　　未来五年，是辽源全面转型的重要攻坚期和战略机遇期。我们要深入贯彻党的十八大精神，以科学发展观统领全局，全面落实市六次党代会提出的目标任务，紧紧围绕加快发展、全面转型，统筹实施开发开放、区域联动、产业升级、文化振兴四大战略，坚持经济建设、政治建设、文化建设、社会建设、生态文明建设五位一体，积极推动工业化、信息化、城镇化、农业现代化进程，加快形成新的经济发展方式，为辽源全面建成小康社会打下决定性基础。

　　经济社会发展的奋斗目标是：经济总量快速增长，现代产业体系基本形成，城市功能品位大幅提升，生态环境质量明显改善，文化软实力显著增强，社会建设取得重要进展，人民生活更加美好。基于对国际国内经济走势的分析和研判，紧密结合我市实际，到2017年，地区生产总值和全口径财政收入力争年均增长25%以上；全社会固定资产投资年均增长30%以上；城镇居民人均可支配收入和农民人均纯收入力争年均增长15%以上；节能减排指标必保完成省下达计划。重点做好以下八个方面工作：

　　一是着力构筑现代产业体系。大力改造提升传统优势产业，加快发展战略性新兴产业和现代服务业。强化投资、消费、出口的协调拉动作用，调整优化经济结构，推进重大项目建设，促进经济可持续发展。更多依靠科技进步、劳动者素质提高和管理创新驱动，加快产业高端化、规模化、集群化、园区化发展。重点打造1个千亿级产业基地、9个百亿级产业集群，辽源经济开发区晋升为国家级开发区，争创全国资源型城市转型示范市。

　　二是着力推进现代城市建设。围绕打造吉林省中南部区域中心城市，完善规划体系，强化规划控制，协调实施老城改造与新城建设，大力提升城市承载功能。完善重大基础设施和相关配套政策，加速产业、要素、人口向中心城区和重点镇集聚，走集约、智能、绿色、低碳的新型城镇化道路。积极开展省级生态城、国家级环保模范城、国家级文明城、国家级园林城、国家级卫生城创建工作，加快建设智慧城市。

　　三是着力增强县域综合实力。坚持以工促农、以城带乡、工农互惠，推动城乡一体化发展。大力推进新型工业化，扶持发展支柱产业、立县企业，打造特色产业园区，培育壮大农业产业化龙头企业。加强农业科技创新和农田水利建设，稳定粮食生产，大力发展现代农业。以农民增收致富为核心，统筹解决好三农问题，加快推进新农村建设。力促县域经济总量大幅跃升，实现县域综合实力在全省升级晋位。

　　四是着力增进人民群众福祉。坚持民生优先，滚动实施惠民工程，让发展成果更多地惠及全市人民。全面完成棚户区、暖房子改造和老旧小区治理工程。深入推进全民创业，扩大社会充分就业，统筹建设城乡社会保障体系。努力办好人民满意教育，不断提高群众健康水平，加快推进基本公共服务均等化。千方百计增加居民收入，切实保障低收入群众基本生活。

　　五是着力加强和创新社会管理。从维护人民群众根本利益出发，加强基层服务管理体系建设，探索具有辽源特色和时代特征的社会管理创新模式。建立健全群众利益保障、社会矛盾化解、公共安全防控、重大决策社会稳定风险评估机制，不断提高社会管理科学化水平。切实加强法制建设，维护社会公平正义，打造平安辽源、法治辽源、活力辽源、和谐辽源。

　　六是着力加快生态文明建设。坚持节约资源和保护环境的基本国策，积极推进绿色发展、循环发展、低碳发展。加快土地节约集约、新能源开发利用、节水型社会等国家级试点建设，形成节约资源和保护环境的空间格局、产业结构、生产方式和生活方式。强化节能减排和污染防治，实施重大生态修复工程，努力建设天蓝、地绿、水净的美丽辽源。

　　七是着力促进文化发展繁荣。用社会主义核心价值体系引领文化建设，凝练城市精神，塑造城市标志，丰富城市色彩，积极推进全民道德工程建设。加快文化事业发展，完善公共文化服务体系，不断丰富群众文化生活。弘扬传统文化，物化、活化特色文化资源，强化公益宣传，营造浓厚氛围，提升公民文化自觉和文化自信。积极培育文化产业，增强文化软实力和竞争力，致力打造特色文化名城。

　　八是着力深化改革扩大开放。加快转变政府职能，切实提高行政效能，建设职能科学、结构优化、廉洁高效、人民满意的服务型政府。更加注重发挥市场作用，不断深化经济、文化、社会、生态建设等重点领域和关键环节改革，充分激发体制机制活力。继续扩大招商引资和对外交流，积极承接发达地区产业转移，促进区域开放合作，努力打造全省发展要素最活跃地区。

　　三、2013年主要工作安排

　　2013年，宏观经济形势复杂多变，发展的任务特别艰巨。党的十八大为我们描绘了全面建成小康社会的宏伟蓝图。市政府将认真按照市六次党代会和市委六届二次全会确定的目标任务，迎难而上，团结拼搏，扎实工作，不断顺应人民群众新期待，努力实现辽源转型发展新跨越。

　　预期目标是：地区生产总值、全口径财政收入增长20%以上，全社会固定资产投资增长30%，社会消费品零售总额增长16%，外贸进出口总额增长20%以上，城镇居民人均可支配收入和农民人均纯收入增长15%以上。城镇登记失业率控制在3.9%以内。人口自然增长率控制在2.3&permil;以内。万元GDP能耗降低率和主要污染物减排量完成省下达指标。重点做好十个方面工作：

　　（一）促进经济持续健康发展。积极应对宏观经济下行压力，按照稳中求进的工作总基调，努力保持加快发展的良好势头。继续扩大投资规模。组织实施投资3000万元以上项目420个，其中亿元以上项目262个，10亿元以上项目49个。重点实施辽西高速、辽长铁路等基础设施项目；东北袜业三期、鑫达钢铁三期等工业项目；辽源国际商贸城、凯旋王国创意园等现代服务业项目；辽河北岸城市综合体、玉圭园城市综合体等房地产项目；国有工矿棚户区改造、矿山湿地公园等民生和生态环境治理项目。找准国家政策切入点，谋划储备一批重大项目。着力拉动消费需求。完善消费政策，推进高端消费本地化、中低档消费便利化，加快培育一批拉动力强的消费增长点。鼓励本地农民进城落户，吸引域外人口来辽购房，活跃二手房交易市场。加快汽车贸易产业园建设，促进汽车等耐用消费品本地采购。打造营业收入30亿元以上的现代服务业商圈。启动2个五星级酒店建设。培育黄牛、梅花鹿、钢材、袜业等专业市场，建立专业网上交易平台。扩大信贷消费，鼓励旅游、健身、养老、家政等服务消费，促进传统服务业提档升级。加快工业提速增效。加大生产运行协调力度，保障水、电、煤、油、气、运等要素配置。全面推进本地企业互采互用，支持跨行业、跨区域协作配套。加强银企保对接，通过债务融资平台、担保融资平台、股权交易平台融资60亿元以上。支持企业扩大再生产，推进在建项目投达产，规模以上工业企业新增30户，规模以上工业增加值达到380亿元，工业增速保持全省领先。用好用足转型政策。借助国家和省支持资源型城市可持续发展的有利契机，充分利用智慧城市、土地节约集约、新能源开发利用等试点政策，促进高端产业向辽源集聚，在资金、项目争取和要素配置方面获得更大支持。

　　（二）提升产业竞争优势。以提高经济增长质量和效益为中心，加大经济结构战略性调整力度，增强经济发展的内生动力和活力，积极构筑现代产业体系。加快传统产业高端化发展。滚动实施重点企业三年技术改造计划，大力推进130个技术改造项目。深入实施科技创新工程，重点推进50项科技攻关、成果转化和创新基金项目，新建20个省市级技术中心。促进信息化与重点产业深度融合，规模以上工业企业生产、管理、创新应用信息技术比例达到60%以上。加快战略性新兴产业规模化发展。重点推进以铝合金板带箔材、锂电池隔膜纸、碳纤维材料为代表的新材料产业；以气风互补发电、垃圾焚烧发电、太阳能发电为代表的新能源产业；以软件产业园为代表的信息产业；以方大锻造为代表的高端装备制造产业。加快优势产业集群化发展。搞好产业布局、项目谋划和定向招商，提高产业关联度、集中度，进一步做大产业集群。高精铝加工、冶金铸造、纺织袜业、汽车零部件、医药健康、矿山装备、建筑机械、软件产业集群占全市工业比重力争达到60%以上。加快产业布局园区化发展。强化园区产业定位、功能分区和规划控制，加速完善园区承载条件，切实提高投资强度、投入产出率和项目摆布科学性。加快富迪纺织、辽河纺织、吉化辽源化工等企业退城进区步伐。推进经济开发区和各工业集中区提档升级，入园企业新增130户以上。加快现代服务业跨越式发展。不断增强现代物流、信息服务、文化旅游等产业服务本地和辐射周边能力，提高服务业占比。重点推进海王医药物流、茵特拉根二期、辽源国际商贸城等项目建设。加强东北无障碍旅游合作，推动旅游产业提速，打造金州鴜鹭湖、南屯基秀水温泉等一批旅游热点。加快构建企业服务体系。深入实施1258大企业培育工程，产值10亿元以上企业达到15户，其中矿业集团、东北袜业园产值达到50亿元，鑫达钢铁产值突破100亿元。启动运行中小企业服务中心，完善产权交易、融资担保、管理咨询、上市培育、互动交流等综合服务功能。探索订单培训、学校代培、联合办学等方式，努力解决企业招工难问题。建立10个国家和省市级创业孵化基地，新增小微企业400户以上。消除各种制度性障碍，保证各种所有制经济依法平等使用生产要素、公平参与市场竞争、同等受到法律保护。

　　（三）加快建设现代城市。按照规划科学、功能完备、特色鲜明、生态宜居的要求，协调推进老城区改造和新城区建设，着力增强中心城区辐射带动功能。完成南部新城六路四桥、四馆、工商学校、公共安全教育基地建设，启动政务中心、技师学院、医药分院建设，居民回迁楼主体封闭。推进寿山组团、北部新城建设，加快辽白经济一体化。完成辽西高速路基桥涵工程，争取辽长铁路、集双高速开工建设，加快四松铁路、大三环前期工作，实施集锡东辽绕越线、辽那公路大修工程，建设辽源城市候机楼。继续推进东辽河、渭津河、大梨树河等堤防加固及中小河流治理工程，加快实施北城区供水系统改造工程，开工建设雨水管网工程。完成市区安北大街中段、安平路东段、前阳街南段、和宁街、新兴北路东段和万源桥、县医院双桥、革新桥建设。深入开展五城联创，争取省级生态城通过达标验收，国家级环保模范城、国家级文明城创建取得重要进展，启动国家级园林城、卫生城创建工作。建设10条精品街路。探索实施符合辽源发展实际的城市管理综合行政执法体制和执法模式。启动运行数字安监、数字消防、数字城管、数字交通系统。

　　（四）全力推进县域突破。坚持以工业化为主导，以城镇化为支撑，促进城乡区域发展协调互动，加速县域经济升级晋位。加快东丰西城区、白云新城建设，启动东辽北城区建设。切实增强城镇综合承载能力，有序推进农业转移人口市民化，全市城镇化率提高1.3个百分点。坚持产城融合，促进产业、要素集聚，不断提高城镇化质量，加快新型城镇化进程。大力支持两县培育支柱产业和立县企业，县域经济主要指标增速高于全省平均水平，年度综合发展指数在全省升级晋位。夯实农业基础，保障农产品供给，粮食产量稳定在40亿斤阶段性水平。大力发展精细种植业、精品养殖业、精深加工业、园艺特产业和乡村旅游业，新建标准化蔬菜大棚1800栋，加快拉拉河蔬菜种植基地建设，改扩新建标准化牧业小区90个，建设6个现代牧业循环经济园区，打造梅花鹿核心产业园区。培育一批省级以上农业产业化龙头企业，提升金翼蛋品、麒鸣牧业、睿康生物等重点企业品牌影响和带动功能。着力构建集约化、专业化、组织化、社会化相结合的新型农业经营体系，新增农村合作经济组织200个以上，建设15个市级以上标准化示范合作社。启动第二批100个省市级新农村示范村和4个样板区建设。

　　（五）认真办好惠民实事。坚持以人为本，切实提高基本公共服务均等化水平，重点实施十个方面惠民工程：一是保障性安居工程。征收房屋1.4万户，建设保障房4.4万套，当年回迁2万户。发放廉租住房租赁补贴1.75万户。完成既有建筑节能改造不低于170万平方米。全面完成老旧小区治理，实现物业管理全覆盖。二是创业带动就业工程。建立创业综合批发大市场和返乡农民工创业园。发放小额担保贷款6000万元以上。劳动者素质培训3.5万人以上，创业培训4000人以上。公益性岗位保持在7500个左右。三是社保扩面提标工程。适时启动厂办大集体职工养老保险接续工作，基本解决国企改制失业保险历史遗留问题。扩大享受财政贴息贷款续保范围，促进非公经济组织参保。继续提高养老、失业保险待遇。四是困难群众救助工程。继续提高城乡低保标准，扩大社会救助范围。资助贫困学生2000人以上。五是促进居民增收工程。严格执行最低工资标准，努力增加居民工资性收入和财产性收入，重点保障低收入群众基本生活。六是公共设施提升工程。天然气用户达到5万户以上。市区24小时供水率达到65%。七是学前教育推进工程。全市新增公办幼儿园20所左右，乡镇公办中心幼儿园实现全覆盖。大力规范和发展民办学前教育。八是医疗服务改善工程。完善全民医保制度，提高城乡居民医保报销比例。建立医疗机构分级诊疗格局，推进全科医生执业方式和服务模式改革试点城市建设。九是社会养老机构培育工程。扩大4050服务7080试点，加快建设城乡社区老年人日间照料中心和城市社区居家养老服务站，改扩建公办社会福利院。十是文化惠民工程。图书馆、群众艺术馆、博物馆、规划科技展览馆、全民健身中心游泳馆投入使用，30个乡镇综合文化站全部达标。继续开展多种形式的群众性文体活动。

　　（六）不断深化改革开放。坚持把发展难点作为改革重点，继续推进行政管理、公共服务、生态建设、城镇化配套等重点领域和关键环节改革。着力培育各类市场主体，为经济发展注入不竭活力。深化土地收储制度改革，创新土地收储交易体制机制。加快投融资体制改革，规范政府融资平台，增加行业融资平台。组建城市基础设施投资公司，新增5个跨行业互保池、2户贷款担保公司，4户小额贷款公司。加快民生银行驻辽分支机构筹建工作。推进三期企业债发行工作。推动金翼蛋品等企业上市步伐。积极承接欧美、东南亚、港澳台和长三角、珠三角等重点地区产业转移，加快融入长吉图开发开放先导区和沈阳、长春两大经济圈。切实增强招商引资的主动性、针对性和实效性，进一步提高资金到位率和项目成功率。坚持引资与引智并举，加速升级辽源人才、产品、技术结构。争取签约落地投资超10亿元项目20个以上，引进域外资金460亿元，实际利用外资2.5亿美元。培育技术、品牌等核心竞争力优势，加强外贸出口基地建设，逐步提高经济发展外向度。

　　（七）积极促进文化振兴。坚持用社会主义核心价值体系凝聚社会共识，大力弘扬开放、创新、诚信、厚德的价值取向和积极向上向善的社会风尚。发展城市文化，挖掘城市内涵，凝练城市精神，广泛征集市标、市歌等城市标识符号。进一步完善公共文化服务，加快城市文体设施、社区和农村文化场站建设，形成覆盖城乡、结构合理、功能健全、实用高效的公共文化服务体系。广泛开展全民健身运动，认真做好十七届省运会场馆建设及各项筹备工作。挖掘整理辽源地域历史文化，开发保护龙首山古城等历史遗迹。整合特色文化资源，大力繁荣文艺创作，搭建文化企业孵化平台，加快发展文化旅游、体育艺术培训、出版印刷、演艺、影视制作、工艺美术六大产业。力争鴜鹭湖旅游度假区、向阳山森林公园、江城动植物园、扎兰芬围影视基地列入国家级3A旅游景区行列。

　　（八）大力推进生态文明建设。推动资源利用方式根本转变，切实提高资源利用效率和效益。全面实施国家土地节约集约、新能源开发利用、节水型社会试点示范城市建设，加快东丰低碳工业园区和东辽金州低碳城镇等项目建设。强化节能减排和环境保护，推进碧水蓝天工程，重点实施金刚水泥脱硝等一批节能减排项目，加快辽源国家矿山公园、矿山湿地公园建设，实施东辽河流域生态保护和杨木水库上游水源地综合治理，逐步提高东辽河出境断面水质质量。加大生态修复整治力度，深入实施《第二个绿化美化辽源大地规划》和三北防护林五期工程，加强森林资源保护和林地用途管制，依法打击乱砍滥伐和蚕食林地行为。强化生态建设目标体系、考核办法和奖惩机制，推进国家级生态镇和省级生态村建设，积极营造爱护生态环境的良好社会氛围。

　　（九）精心维护社会和谐稳定。深入推进社会管理创新，不断完善党委领导、政府负责、社会协同、公众参与、法治保障的社会管理体制。全面提升社会管理水平，重点抓好行政服务中心、公共资源交易中心、矛盾联合调处中心、应急监控指挥中心、社区便民服务中心、农村三资代理中心建设。实施领导包案和积案化解攻坚，积极满足群众合理诉求，妥善处理非正常上访。探索信访预约、集中会办、公开听证等形式，把矛盾化解在基层和萌芽状态。进一步加大社区建设投入，延伸社区服务功能，发挥志愿者组织积极作用，提高基层组织服务群众能力。建立覆盖城乡的全方位、立体化治安防控体系，依法防范和打击各类违法犯罪活动，保障人民群众生命财产安全。严格落实安全生产责任制和责任追究制，坚决防止较大以上事故发生。健全食品药品安全监管体系，加大整治力度，确保群众饮食用药安全。积极开展六五普法。加强气象、地质灾害预报预警，完善防灾减灾预案，构建实战化的应急指挥体系。

　　加强国防动员和后备力量建设，积极争创全国双拥模范城。坚持计划生育基本国策，进一步提高出生人口素质。统筹做好民族宗教、外事侨务、档案、方志、妇女儿童、老龄、残疾人等各项工作。

　　（十）切实加强政府自身建设。新形势、新任务要求我们必须把自身建设摆到更加突出位置，努力建设人民满意政府。要进一步加强能力建设。深入学习贯彻党的十八大精神，系统掌握经济发展、城市建设、社会管理等方面知识，切实提高干部理论素养、政策水平和业务能力。要不断解放思想，更新理念，与时俱进，创造性开展工作，牢牢把握发展主动权。要努力在创新实践中提升能力，在破解难题方面增强本领，坚持用发展的办法解决前进中的问题和矛盾。要进一步加强效能建设。认真落实一岗五责，切实增强公信力、执行力，积极构建务实高效的行政管理体制。坚持科学民主决策，大力推进依法行政，继续完善政府议事规则，健全决策会商、评价反馈机制。简化办事程序，优化发展环境，强化政府绩效考核，建设数字监察系统。积极回应百姓诉求，高度重视社情民意，自觉接受人大、政协、人民群众和新闻舆论监督。要进一步加强作风建设。牢固树立以人为本、执政为民理念，倾听群众呼声，关心群众疾苦，认真解决人民群众反映强烈的突出问题。勇于担当，敢于负责，务实高效，说实话，办实事，求实效。多到矛盾困难集中、群众意见最多的地方调查研究，多深入基层和群众之中解决重点难点问题。改进会风文风，力戒空话套话，少开会、开短会，少发文、发短文。着力整治庸懒散奢等不良习气，确保政令畅通。要进一步加强廉政建设。全面落实党风廉政建设责任制，完善工程招投标、土地招拍挂、国有资产管理、大额资金使用等监督制度。厉行勤俭节约，压缩行政开支，减少各类庆典活动。完善监察和审计体制，严肃查处违法违纪案件，努力建设清廉政府。健全权力运行制约和监督体系，坚持改革限权、依法确权、科学配权、公开示权、全程控权，让人民监督权力，让权力在阳光下运行。

　　各位代表，辽源已经站在新的历史起点上。让我们在党的十八大精神指引下，在市委的坚强领导下，团结带领全市各族人民，深入贯彻落实科学发展观，解放思想，振奋精神，拼搏进取，扎实工作，为加快建设富裕文明、充满活力、幸福和谐的新辽源而努力奋斗！
